# General Nursing Council for Scotland

**Published:** 1921-01-29

**Authors:** 


### General Nursing Council for Scotland.

### Two Important Decisions.

A MEETING of the General Nursing Council for Scotland was held on Wednesday, January 12. Captain Charles B. Balfour, C.B., was in the chair, and thirteen members of Council were present.

The Registrar reported that no reply had been received from the Scottish Board of Health to his letter to the Board of December 8 in regard to the Board's interpretation of Rule 20 (3) (*a*) of the Council's Draft Rules in regard to existing nurses—this being the rule under which the Board maintained that they were entitled to insist on existing nurses holding their fever nursing certificate being put on the General Register.

Correspondence with the English and Irish Councils in regard to the points outstanding between the Scottish Council and them was considered.

ANOTHER SUPPLEMENTARY REGISTER.

A letter was submitted from the General Board of Control for Scotland, along with a statement containing information regarding the numbers and training of nurses at present employed in institutions for mental defectives in Scotland. In view of this information and on the recommendation of the Board of Control, the Council resolved to add to their draft rules a provision prescribing a supplementary part of the Register for nurses trained in the care of mental defectives. It was resolved that this should be a separate supplementary part, and not a subdivision of the Mental Nurses' Register as provided in the English and Irish rules.

EXISTING NURSES WITHOUT HOSPITAL TRAINING.

The Council again considered the draft rules for existing nurses, and, in view of the difficulty of obtaining and weighing special evidence of adequate knowledge and experience where none of such knowledge and experience has been obtained in a hospital or institution recognised by the Council, the Council unanimously agreed to delete Draft Rule 20 (3) (*d*), which provided for the Council accepting such special evidence where an existing nurse applying for admission to the General Register had no hospital training.

Dr. Fraser, Convener of the Education and Examination Committee, submitted an interim report on the work of that committee.

